# TB and environmental health: global research trends and opportunities for future studies in Africa

**DOI:** 10.5588/ijtldopen.26.0213

**Published:** 2026-11-13

**Authors:** N. Naidoo, R. Naidoo, M. Bulani, E. Spooner, N. Jafta, T. Kapwata, B. Shezi, D.M.K. Kironde Kibirige, N.E. Mvubu, N. Moodley, C. Xiong, C.Y. Wright

**Affiliations:** 1 Climate Change and Health Research Programme, Environment and Health Research Unit, South African Medical Research Council, Durban, South Africa;; 2 Discipline of Occupational and Environmental Health, School of Medicine, College of Health Sciences, University of KwaZulu-Natal, Durban, South Africa;; 3 Climate Change and Health Research Programme, Environment and Health Research Unit, South African Medical Research Council, Pretoria, South Africa;; 4 HIV and other Infectious Diseases Research Unit (HIDRU), South African Medical Research Council, Durban, South Africa;; 5 Environmental and Geographical Science, University of Cape Town, Cape Town, South Africa;; 6 Discipline of Medical Microbiology, School of Laboratory Medicine and Medical Science, Nelson Mandela Medical School, University of KwaZulu-Natal, Durban, South Africa;; 7 Department of Medical Biosciences, Faculty of Natural Sciences, University of the Western Cape, Cape Town, South Africa;; 8 Data Management and GIS Unit, KwaZulu-Natal Department of Health, Durban, South Africa;; 9 Center for Human-Environmental Systems (CHES), Department of Civil and Environmental Engineering, Villanova University, Villanova, PA, USA;; 10 Department of Geography, Geoinformatics and Meteorology, University of Pretoria, Pretoria, South Africa.

**Keywords:** tuberculosis, air pollution, bibliometrics, environmental exposure, health equity, LMICs, temporal trends

## Abstract

**BACKGROUND:**

TB, a major global public health concern, remains one of the most widely studied infectious diseases. However, TB control strategies do not consider environmental conditions such as heat and air pollution as major factors in policy implementation. This study presents a bibliometric analysis exploring the impact of heat and air pollution on TB burden.

**METHODS:**

Using PubMed, Scopus, World of Science Core Collection, and Ovid MEDLINE databases, 124 studies were extracted, all of which related to the effects of environmental factors on TB transmission and epidemiology. A bibliometric analysis was carried out on Bibliometrix and VOSviewer.

**RESULTS:**

There is a disproportionate representation in the global discourse on environmental factors that contribute to TB transmission and epidemiology. China had the highest volume of research outputs and low- to middle-income countries were largely absent. Seasonality and air pollution were frequently studied, with time-series studies and meteorological modelling emerging as niche methods.

**DISCUSSION:**

Global research efforts on linking TB transmission and epidemiology to environmental factors have been established, but more effort is needed to characterise these relationships in different regions. Future research should explore the mechanistic/causal pathways of environmental TB risk, particularly with increasing air pollution and climate change affecting developing nations.

TB remains one of the leading causes of death from an infectious disease globally. In 2024, Africa had ∼2.62 million TB cases, about one quarter of the global burden (10.7 million cases), and the highest TB incidence rate at 207 per 100,000 population, compared with the global average of 131 per 100,000 cases.^1^ Africa has a 428,000 TB cases among people living with HIV and a rate of 34 per 100,000, far exceeding the global rate of 7.6 per 100,000.^1^ Thus, TB research focuses on advancing public health measures and reducing transmission, preventing illness, and ultimately saving millions of lives.

Heat amplifies the spread and changing endemicity of infectious diseases, changes weather patterns amplified by climate change, increases vulnerability to air pollution, and drives population displacement.^2^ There are an increased number of studies exploring environmental/climatic factors that play a role in exacerbating the TB burden.^3^ However, while environmental health research is expanding, a cohesive understanding of how environmental factors, influenced by climate change, intersect with TB burden remains unclear. It remains difficult to prove a definitive association between some environmental factors and TB epidemiology or even transmission trends in low-income communities. Much of the existing evidence focuses on indoor environmental conditions, including ventilation, crowding, and household air pollution, all of which have been strongly associated with increased risk of TB transmission.^4^

There is a growing body of literature on TB, totalling 263,234 articles published between 1849 and 2020.^5^ Most TB articles come from the subject area of ‘medicine’ (88.31%), followed by subject areas such as ‘immunology and microbiology’ (7%). Studies from ‘environmental science’ make up 1% of this literature and has not been properly characterised.^5^ Notably, the environmental science subset literature has not undergone the same rigorous structural and content-based analyses as the full body of TB literature, resulting in a gap in understanding its internal dynamics. The aim of this study was to map the global trends in research exploring environmental/climate variables and TB.

## METHODS

We conducted a literature search to build the foundation for our bibliometric analysis. The selection process ensured an exhaustive coverage of studies exploring environmental studies of TB from 1990 to 2025. The literature search was guided by Arksey and O’Malley framework and the Joanna Briggs Institute^6,7^ (Figure 1).

**Figure 1.**
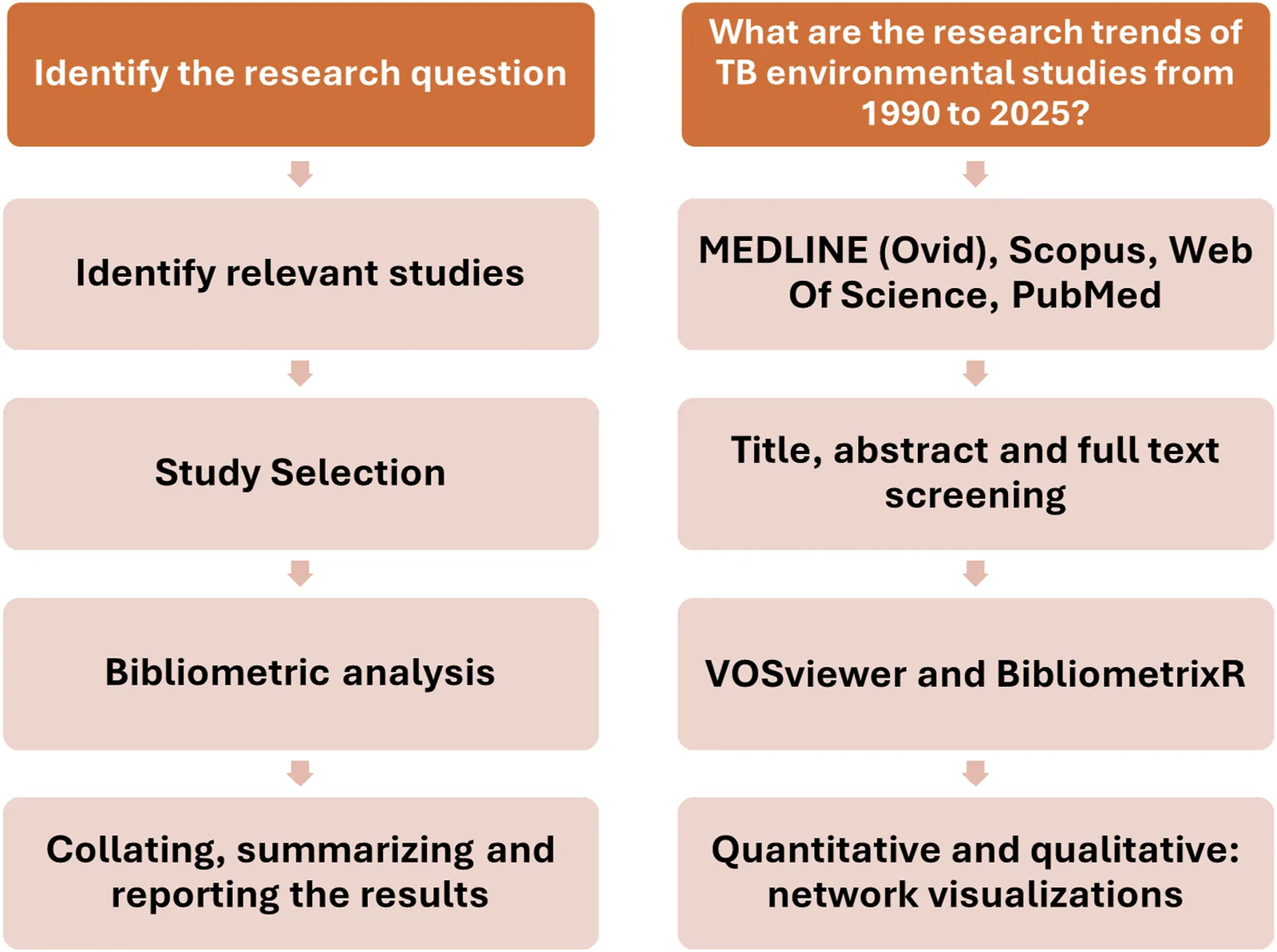
The phases of the bibliometric study of the effects of environmental factors and climate change on TB disease dynamics.

The search strategy was designed to capture all relevant publications via four databases: PubMed, Scopus, Web of Science Core Collection, and EBSCO. The search was executed via keywords related to the three main domains: TB, environment/climate, and health outcomes (Supplementary Data Table 1). The query was tailored to the title, abstract, and keyword fields (Supplementary Data Table 1) and inclusion and exclusion criteria are given in Supplementary Data Table 2.

The retrieved records from all four databases were exported in bibtex format. All records were imported into a single bibliographic management software (Rayyan). Duplicates were removed across the combined dataset. Following deduplication, an initial screening was performed to filter out records irrelevant to human pulmonary and disseminated TB or where the focus on environmental variables was non-existent or minimal. The abstract and title screening was done by five authors (NN, MB, RN, ES, and NJ) to ensure consistent and unbiased data extraction. Conflicts were resolved through a consensus meeting. For articles where no consensus was reached, a senior reviewer (CYW) served as an arbiter. The full text screening was carried out by NN and CYW.

The bibliometric analysis was done via the Bibliometrix R package (statistical analysis and performance metrics)^8^ and VOS viewer (network visualisation and mapping).^9^

### Ethical statement

No ethical approval was required as there were no human participants.

## RESULTS

The search criteria yielded 1,096 articles, and after full text screening, 124 articles were chosen to be included in the final bibliometric analysis. Two articles in Russian and two in Chinese were excluded as they were not available in English (Supplementary Data Figure S1).

### Country-specific scientific production and global collaborative networks

China had the highest number of studies (n = 70), followed by the USA (n = 6), South Africa (n = 6), and Japan (n = 5) – see Figure 2A. Several countries including parts of Africa, Asia, South America, the Middle East, and Europe reported few studies (n = 1–3), with seven global studies not featured on the map. China was the most prolific author contributor (n = 427) (Supplementary Data Table S3; Figure 2B). The USA ranked second (n = 77) (Supplementary Data Table 3), followed by the UK (n = 27).

**Figure 2.**
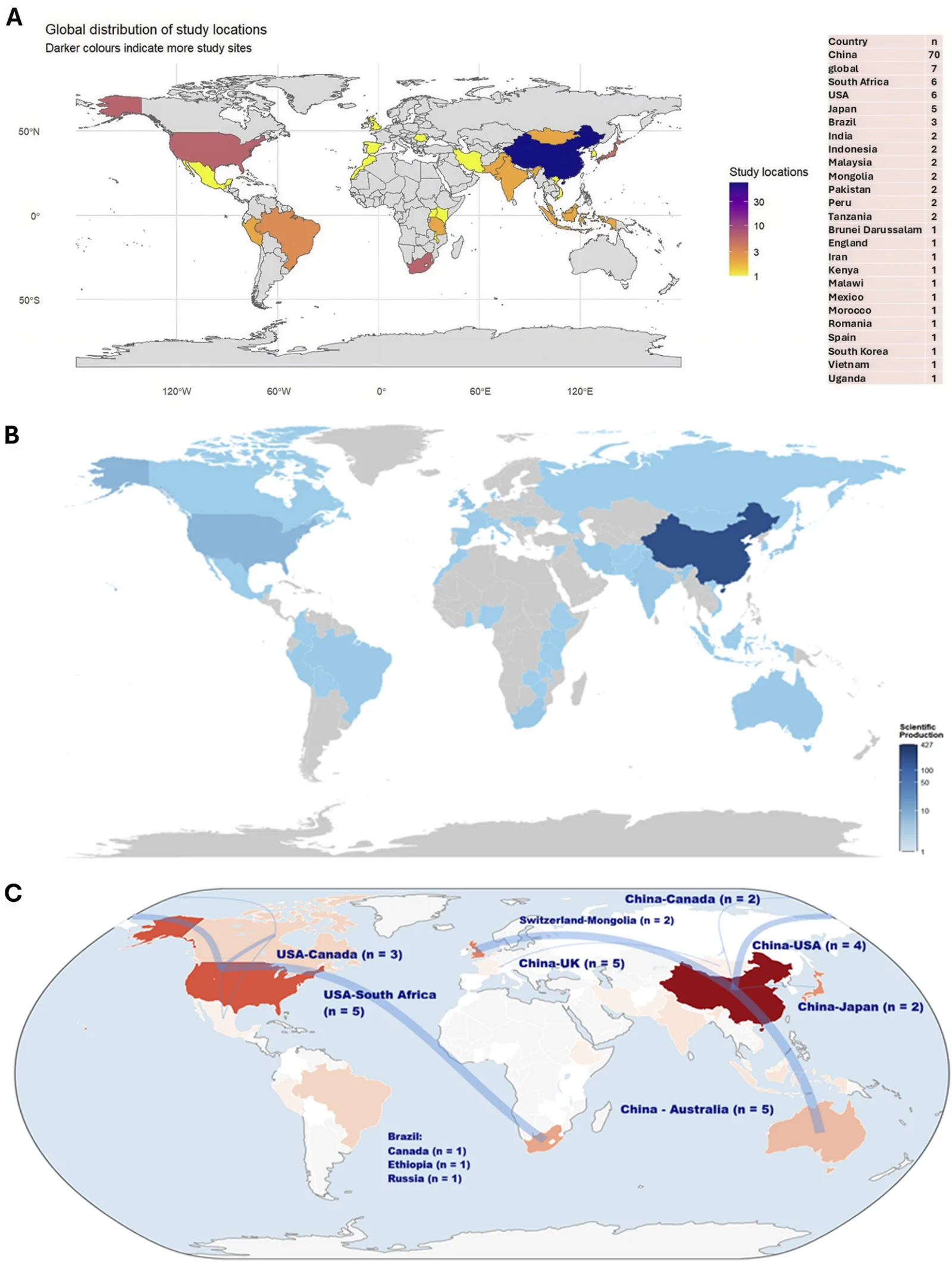
**A:** Global distribution of study locations from the screened studies; countries are shaded according to the number of study locations within a country, with darker colours indicating higher counts; **B:** Map showing the frequency of authors per country reflecting the global scientific production datasets; **C:** Country collaborative map, showing the countries from which authors have collaborated within the search field.

The country collaborative map presents the international research partnerships, with countries shaded by contribution intensity and curved arrows indicating co-authorship links and their frequency (Figure 2C). China was the central hub of collaboration, showing the strongest connections with the UK (n = 5), Australia (n = 5), the USA (n = 4), Japan, and Canada (each n = 2). Additional bilateral collaborations include USA–South Africa (n = 5), USA–Canada (n = 3), and Switzerland–Mongolia (n = 2). A small number of collaborations are noted from Brazil, Ethiopia, and Russia (each n = 1). Australian researchers did not conduct studies within Australia (see Figure 2A).

### Descriptive bibliometric analysis

Databases returned publications from 2004 to 2025, sourced from 66 journals (Figure 3). The average age of the literature is 6 years, indicating that much of the literature is relatively recent. The documents show an average of eight citations per publication. On average, each document involves ∼8.5 co-authors. The dataset includes 277 author keywords. Annual publications increased over time, with modest early output and accelerated growth from the mid-2010s, reflecting rising interest and field consolidation (Figure 4). Average citations peaked earlier and have since declined, consistent with field maturation and shorter accumulation time for newer studies (Figure 4). The growth curve and publication counts indicate a shift from emergence to rapid expansion, approaching a peak and expected decline.

**Figure 3.**
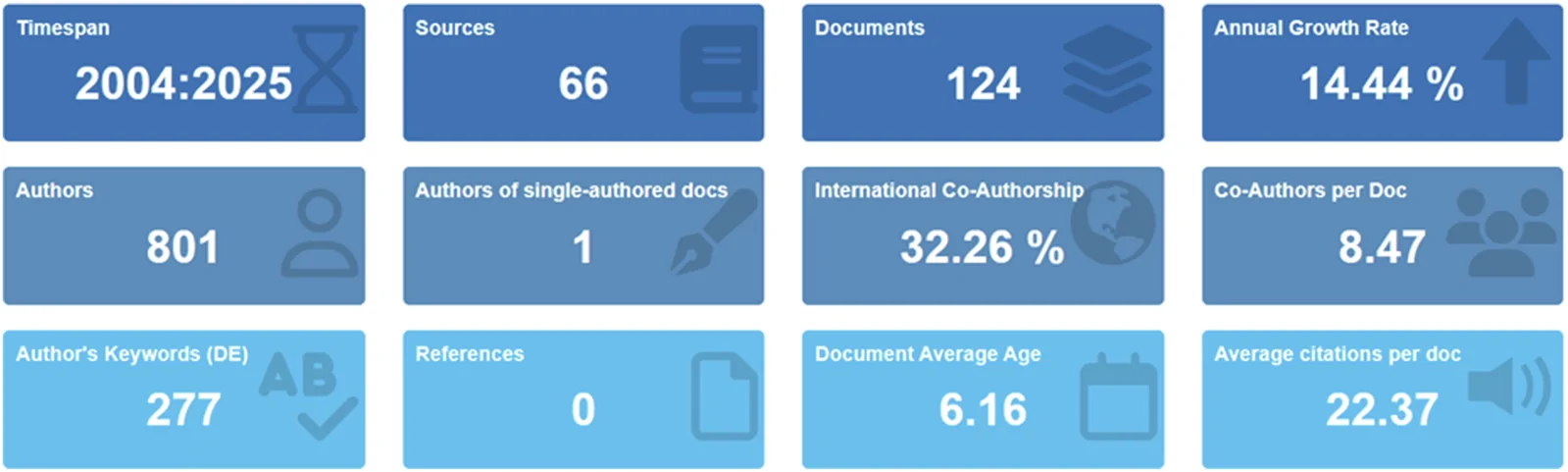
The main characteristics output of the total literature screened from the databases and analysed on Bibliometrix.

**Figure 4.**
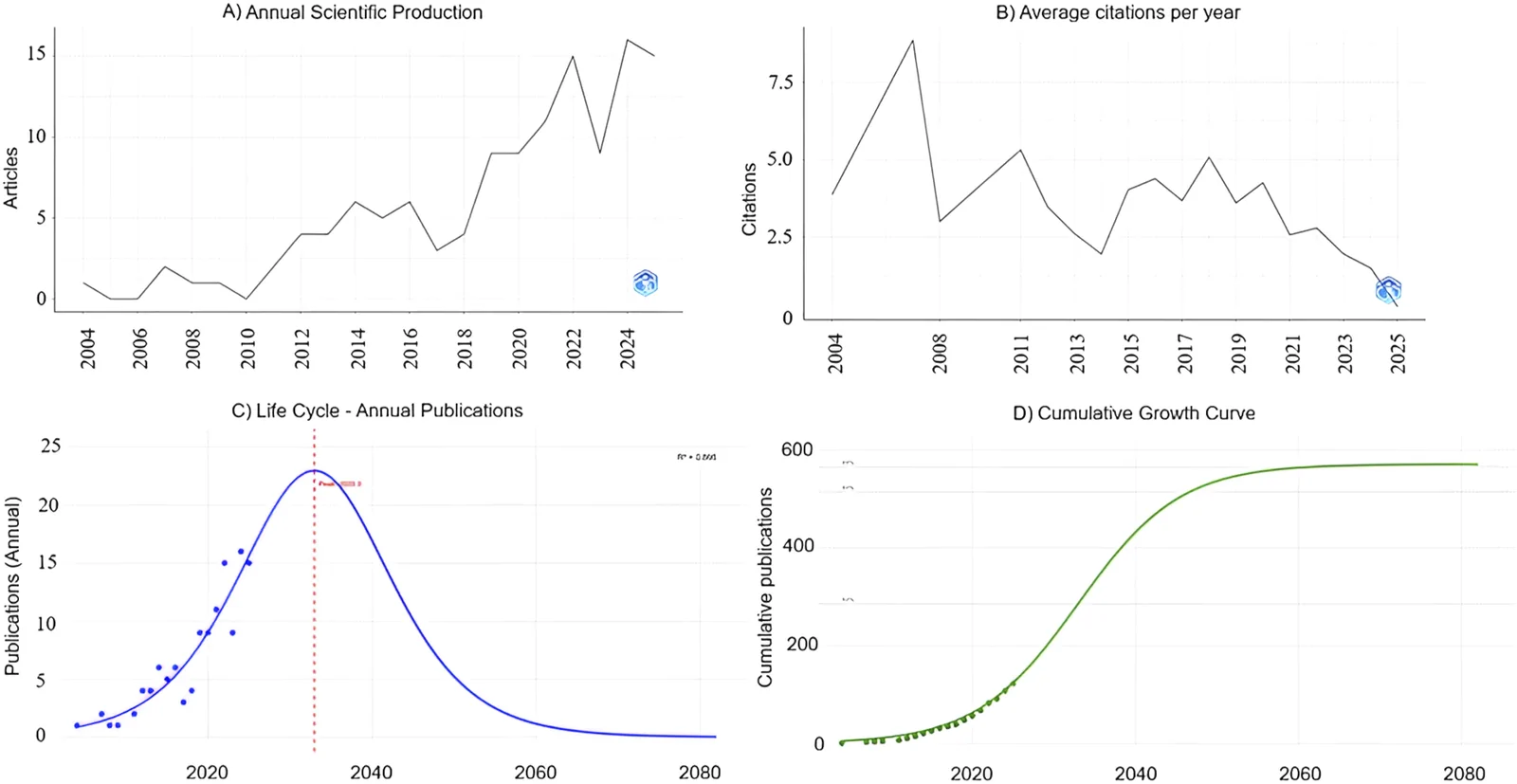
Bibliometric trends and growth dynamics of the research field (2004–2025). **A:** Annual scientific production showing the number of articles published per year; **B**: Average citations per year, indicating changes in citation impact over time; **C:** Life-cycle model of annual publications, with observed data points and a fitted curve illustrating the developmental trajectory of the field; the dashed vertical line marks the estimated peak phase; **D**: Cumulative growth curve of publications, showing the long-term expansion and projected saturation of the literature.

China is the leading contributor to highly cited research in this field, highlighting its strong influence on global discourse around TB and environmental factors, followed by the USA, whose smaller output is relatively highly cited (Supplementary Data Figure 2). China dominates both single-country and multi-country publications, while the USA demonstrates a balanced contribution across both, reinforcing its role as a key contributor and collaborative hub (Supplementary Data Figure 3).

### Trends in publication sources reporting environmental links to TB per country

The Sankey diagram illustrates connections between countries, journals, and authors, highlighting the interdisciplinary and internationally collaborative nature of the field, led predominantly by China with contributions from the USA and other countries (Figure 5). Despite this, low- and middle-income countries (LMICs) with high TB burden remain underrepresented. Major journals (BMC Public Health, Frontiers in Public Health, BMJ Open, PLOS One) are largely linked to Chinese authors, with more limited contributions from India, Japan, South Africa, and the UK (Figure 5).

**Figure 5.**
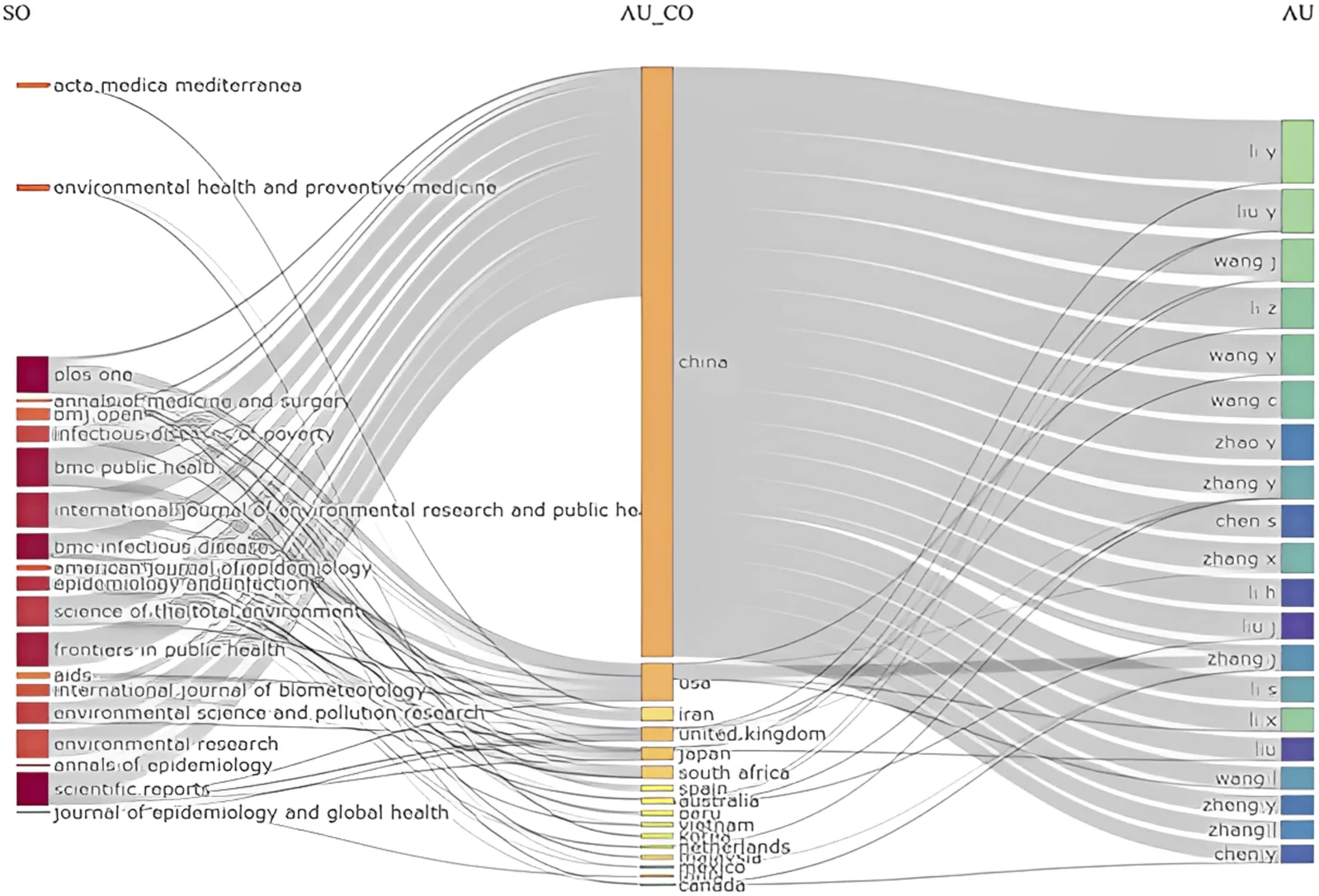
This three-field plot illustrates the connection between sources, countries, and authors. SO = source; AU_CO = author country; AU = author.

### Global impact of publications from authors from each country

The Bibliometrix analysis identified authors with the greatest citation impact within the retrieved dataset. The H-index, calculated from dataset-specific citations, reflects local rather than global impact (Supplementary Data Figure 4). It represents the number of publications (H) with at least H citations.

### Keyword analysis

The VOSviewer network visualisation shows keyword co-occurrences across TB, air pollution, and climatic research (Supplementary Data Figure 5).

The thematic map shows that TB research is primarily concentrated on mature, central themes such as seasonality, pulmonary TB, and epidemiology (Supplementary Data Figure 6). Environmental factors and risk assessment appear as weakly developed, peripheral themes, indicating limited representation in the literature. Few niche themes are evident, with clinical and epidemiological topics predominating.

### The top 20 most cited articles

The most highly cited publications focused on vitamin D status, while the remaining studies show consistent patterns linking TB incidence and transmission to environmental, seasonal, and socio-spatial factors (Supplementary Data Table 4). Several studies report positive associations between air pollution and increased TB risk,^10–16^ alongside evidence on the role of crowding and ventilation in public transport in transmission.^17^ Seasonal trends and spatial clustering of TB cases and mortality are also reported.^18–21^ Studies from China examine air pollution, spatial patterns, and meteorological drivers of TB.^11–13,20,22,23^ Studies from South Africa focus on seasonality and public transport.^17,21^ Tanzania reports nutrition and host factors and the USA on biological mechanisms linking air pollution to TB.^10,14,15^

## DISCUSSION

The current literature search showed that before 2004, there were no significant published contributions on the impacts of environmental factors on TB burden and epidemiology. The delayed emergence of this research topic may reflect conceptual and technological constraints within earlier epidemiological data. It may also indicate the earlier emphasis of studies focusing on biomedical and socio-economic aspects of localised TB epidemics such as HIV co-infection, poverty, and overcrowding.^24–26^

The early 2000s marked a turning point, driven by advances in satellite-derived and ground-based climate and air pollution data,^27,28^ improved geospatial analytical tools, and the growing recognition of climate change as a global health threat.^29^ Research using more advanced data science has been more inclined towards malaria and other vector-borne diseases.^30^ Since the life cycles of vectors such as mosquitoes and the development of parasites are so sensitive to rainfall and temperature, these infectious diseases are more prominently studied.^31,32^ The post-2004 expansion of interdisciplinary science linking environmental science, public health, and infectious disease epidemiology creates a much-needed intellectual space for this field to emerge as a distinct area of inquiry within the traditional TB research space.

The global patterns of research collaborations in TB-environment interactions highlight some global connections. This analysis shows that countries with a relatively lower TB burden but strong research infrastructure, such as the USA and the UK, are among the more productive contributors to environmental and climate-related TB research. The well-established research institutions, stable funding mechanisms, advanced environmental monitoring systems, and access to high-performance analytical tools drive research in these countries. In 2023, China’s ∼6.8% of the global TB burden and significant drug-resistant TB prevalence^33^ warranted national investments in environmental health and infectious disease research.^34^ Chinese cities account for more than half of the world’s top 10 scientific research hubs, with Beijing retaining its position as the leading global science city.^35^ Rapid industrialisation has intensified air pollution challenges, creating both an urgent public health need and a growing research environment for examining the links between environmental exposures and respiratory diseases.^35^ China’s established leadership in air pollution science, alongside strong interdisciplinary collaboration between environmental scientists and public health researchers reinforces its high publication output in studies linking environmental exposures to TB. Despite bearing a disproportionate share of the global TB burden, many other LMICs contribute relatively few studies. Structural barriers such as limited research funding, inadequate environmental monitoring, and restricted access to high-resolution exposure data likely constrain TB–environmental research in LMICs. African countries follow the World Health Organization interim level targets,^36^ that are only stepping stones towards stricter air quality guidelines.^37^ More studies in LMICs with a high TB burden could establish stricter air quality guidelines and inform strategies to lower health risks. More globally representative evidence-based research will ensure that future research informs equitable, locally appropriate environmental strategies and climate adaptation policies embedded into TB control programmes.

The analysis did not show many collaborations within Africa. South Africa, for example, has a tertiary education policy that promotes expanded research partnerships with the African continent and the Global South.^38^ While South African universities have increased the number of countries they collaborate with, including Brazil, Russia, India, and China, research partnerships remain largely Eurocentric and oriented toward the Global North.^38^ This reinforces geographic and epistemic imbalances, limiting the diversity of perspectives and locally relevant insights.^38,39^ Overcoming these obstacles will be critical for fostering more balanced knowledge production, enabling high-burden and resource-constrained countries to contribute meaningfully to the global evidence base and to co-shape interventions that are contextually appropriate.

The keyword analysis reveals a research landscape characterised by a focus on seasonality and epidemiology. However, while the ‘when’ of TB fluctuations is well-documented, the ‘why’ remains marginal. This suggests that the field is currently more descriptive than mechanistic. Research has yet to fully integrate complex environmental risk variables such as specific indoor air chemistry or precise ecological niches into standard TB control frameworks, and basic epidemiological data must evolve into proactive environmental risk management. Particulate matter (PM) is increasingly recognised not just as a respiratory irritant but as a potential factor that impairs alveolar macrophage function, thereby increasing susceptibility to *Mycobacterium tuberculosis*.^15,40,41^ The interconnection between ‘temperature’, ‘air pollutants’, and ‘associations’ in our keyword mapping suggests that the synergy between climate and pollution is becoming the primary focus for understanding TB transmission dynamics.

The analysis appears to be missing keywords related to socio-economic and demographic factors (e.g., poverty, income, urbanisation, population density, and housing conditions), co-morbidities and health determinants (e.g., HIV, malnutrition, and smoking), and adaptation or intervention strategies (e.g., mitigation, policy, and public health measures). These gaps suggest limited attention to how social vulnerability and individual-level risk factors interact with environmental exposures to influence TB outcomes. Additionally, there seems to be minimal representation of longitudinal or experimental studies, behavioural aspects, and indoor environmental conditions (ventilation, crowding) that could modulate exposure and transmission. This indicates a need for research that integrates environmental, biological, and social determinants, as well as intervention-focused studies to inform policy and public health strategies.

The most influential of the top 20 cited articles were based on vitamin D deficiency. Early highly cited studies established a foundational link between sunlight exposure, vitamin D status, and TB notifications, reinforcing the biological plausibility of seasonal TB patterns. TB cases peak in spring and early summer and are lowest in autumn, indicating seasonal fluctuations in vitamin D linked to sunlight exposure.^42,43^ Vitamin D deficiency was common among TB patients and consistently associated with increased TB risk, regardless of HIV status, suggesting a fundamental role in immune defence against TB.^42^ Importantly, TB patients not only had significantly lower vitamin D levels than their healthy contacts but also failed to show the normal seasonal rise in vitamin D, indicating abnormal vitamin D metabolism and heightened susceptibility.^43^ These findings position nutritional and immunological pathways as central mechanisms through which environmental conditions may influence TB susceptibility.

Exposure to nitrogen dioxide (NO_2_) and carbon monoxide (CO),^10^ PM_2.5_ and PM_10_, sulphur dioxide (SO_2_), and ozone (O_3_)^11^ is associated with increased TB incidence, outpatient visits, and even drug-resistant TB.^13^ Mechanistic evidence demonstrates that air pollutants impair key immune responses, weakening the body’s ability to control *M. tuberculosis*.^14^ In addition, exposure to biomass smoke, especially among women and children, further increases susceptibility to respiratory infections, including TB.^16^ Together, this evidence positions air pollution as both a direct and indirect driver of TB through immune suppression.

TB transmission is strongly influenced by the built environment, social conditions, and climate. High-density, poorly ventilated spaces such as public transport systems have been identified as critical transmission settings, with studies in South Africa showing that minibus taxis sustain TB spread due to overcrowding and inadequate airflow.^17^ Seasonal and meteorological factors also play an important role, with TB incidence often peaking in winter or early spring in Japan, with the strength of seasonality declining over time^19^ and higher TB burden observed in cold, dry, and windy conditions in China.^20^ Yet, a study in China showed similarities in TB burden during winter and summer in the presence of air pollution exposure.^11^ Along with employment, population density, and air quality, it is important to consider the biological effects, such as seasonal variation in immunity, and behavioural factors, such as increased indoor crowding in the regions where studies took place.^22^

The robustness of the bibliometric findings is directly contingent upon the completeness and quality of the available literature and the precision of the search strategy employed. Several limitations inherent to this quantitative method must be acknowledged. First, the search strategy, regardless of its comprehensiveness, carries the risk of omitting relevant articles if key terms are not present in the title, abstract, or keywords. This issue is compounded when authors of relevant articles, particularly those focusing on endemic populations, use generalised language instead of specific terms. Second, bibliometric analysis measures publication activity and impact rather than methodological rigour. The exclusion of non-English literature, particularly the identified Russian and Chinese articles, may have omitted critical localised knowledge from regions with unique ecological profiles and long histories of TB research. There is an absence of geographic data across high-burden regions in sub-Saharan Africa and South America. The environmental drivers in a high-altitude South American community or a humid sub-Saharan tropical zone differ fundamentally from those in industrial Asia. This bias creates an evidence base that fails to represent specific environmental vulnerabilities of the most affected populations.

TB remains a preventable and treatable infectious disease.^44^ However, the control of TB transmission is increasingly challenged by the emergence and spread of antimicrobial resistance, as well as the transmission of highly virulent strains in vulnerable populations (especially low-income communities).^45–47^ Despite effective treatment regimens, transmission and drug resistance underscore the need for understanding TB epidemiology. The resultant TB epidemiology stemming from the transition from TB infection to active disease in response to environmental drivers is critical but not yet well understood.^48^ While a substantial proportion of the global population harbours TB infection, the environmental determinants that precipitate reactivation remain poorly characterised.^49,50^ Uncovering the mechanistic pathways through which environmental exposures such as air pollution and heat influence TB activation and transmission is essential. Advancing research at the intersection of TB and environmental health has important implications for both policy and practice. Generating robust evidence on the role of environmental risk factors can strengthen advocacy for improved air quality standards and support the integration of environmental interventions into TB control programmes.^51,52^ This is particularly relevant in LMICs undergoing rapid industrialisation, but is also relevant to the global North, where industrialisation is at a profound height and where industrial activity is increasing pollutant exposures that may exacerbate TB risk.

There is contrasting evidence of rising temperatures increasing transmission patterns, with emerging evidence of TB risk at higher temperatures.^53^ Evidence from warmer climates and environmental and climate considerations in TB prevention and control frameworks ensure resilient, context-specific, and forward-looking public health responses. A TB–environmental research agenda has the potential to bridge critical knowledge gaps, inform multisectoral policy action, and enhance the effectiveness of TB control efforts in both high-burden and emerging settings.

The growth of this research area has not influenced policy adoption.^54^ The lack of attention to the TB burden–environment link in several low-income, high-TB-burden countries calls into question whether the foundational associations have been properly established in different regions of the world. Since the foundational associations are established in some populations, future work can shift toward mechanistic studies, high-resolution exposure assessments, longitudinal study designs, and intervention-oriented research. To advance this field, we require context-specific evidence to inform policies and interventions that meaningfully address the environmental drivers of TB. The thematic map highlights that TB–environmental studies lack a foundational theoretical framework. The field requires environmental risk indices or integrated bio-meteorological frameworks that can serve as a common thread for transdisciplinary research. Without these stabilising concepts, the field risks remaining a collection of fragmented observations rather than a cohesive scientific discipline. Current literature is heavily saturated with broad associations, noting that TB cases rise when PM_2.5_ levels peak. However, to move the needle on public health policy, research must shift from correlation to causality. We need studies that move beyond general ambient air data to investigate specific biological pathways, such as how specific pollutants alter the respiratory microbiome or the precise ‘lag effect’ of temperature and humidity on *M. tuberculosis* survival. By focusing on these specialised sub-themes as highlighted in our analyses, researchers can provide the mechanistic evidence required to justify urban planning changes or industrial air filtration mandates in high-risk zones.

## CONCLUSIONS

With ∼10.7 million people contracting TB in 2024 alone, TB remains one of the world’s most formidable public health challenges.^55^ Clinical and socio-economic factors have been the traditional focus of risk factors that lead to TB transmission in populations, and not much emphasis has been placed on the role of the environment.^56^ However, as the global climate shifts towards warmer and more unpredictable weather conditions and rapid urbanisation causes poorer air quality, understanding how meteorological factors and air pollutants influence TB susceptibility and transmission has become paramount.^57^

Environmental TB research is a dynamic and rapidly maturing field, characterised by a steady annual growth rate and a shift toward sophisticated statistical modelling. Our findings underscore a profound geographic imbalance, driven by high-intensity research into air pollution and climatic variability in China, while high-burden regions in Africa and South America remain significantly underrepresented. Furthermore, while the links between seasonality, air quality, and TB are well-documented, ‘risk assessment’ and ‘environmental determinants’ remain underdeveloped as niche themes. Research must prioritise international collaborations that empower researchers in high-burden LMICs. Strengthening these global networks is essential to developing localised, climate-resilient public health strategies that address the environmental drivers of TB in the world’s most vulnerable populations.

## Supplementary Material
