# Supplementary material for: TB and environmental health: global research trends and opportunities for future studies in Africa
Source: IJTLD Open. 2026 Nov 13;3(11):685–94. doi: 10.5588/ijtldopen.26.0213 (PMC13764020; doi:10.5588/ijtldopen.26.0213)
Supplement: Supplementary file 1 [file ijtldopen26-0213_supplementarydata1.pdf]

Supplementary data

PUBMED (National Library of Medicine, NIH)

Uses **MeSH** and **title/abstract** fields:

```
(
"Mycobacterium tuberculosis"[Mesh] OR "Tuberculosis"[Mesh] OR "Tuberculosis, Pulmonary"[Mesh]
OR "Mycobacterium tuberculosis"[tiab] OR tuberculosis[tiab] OR TB[tiab]
OR "pulmonary tuberculosis"[tiab] OR "extrapulmonary tuberculosis"[tiab]
OR "disseminated tuberculosis"[tiab]
OR "drug-resistant tuberculosis"[tiab] OR "drug resistant TB"[tiab]
OR "multidrug-resistant tuberculosis"[tiab] OR "MDR TB"[tiab] OR "MDR-TB"[tiab]
OR "extensively drug-resistant tuberculosis"[tiab] OR "XDR TB"[tiab] OR "XDR-TB"[tiab]
OR "rifampicin-resistant TB"[tiab] OR "RR-TB"[tiab]
OR "tuberculous meningitis"[tiab] OR "miliary tuberculosis"[tiab] OR "TB meningitis"[tiab]
OR MTB[tiab] OR "Mycobacterium tuberculosis complex"[tiab]
)
AND
(
"Climate Change"[Mesh] OR "Environmental Exposure"[Mesh]
OR "climate change"[tiab] OR "climate variability"[tiab]

temperature[tiab] OR "temperature variation*"[tiab]
OR heat[tiab] OR cold[tiab]
OR heatwave*[tiab] OR "heat wave*"[tiab] OR "extreme temperature*"[tiab]
OR "extreme weather*"[tiab] OR "weather variability"[tiab]
OR season*[tiab]

"air pollution"[tiab] OR "air quality"[tiab]
OR "particulate matter"[tiab] OR PM2.5[tiab] OR PM10[tiab]
OR ozone[tiab] OR O3[tiab] OR NO2[tiab] OR "nitrogen dioxide"[tiab]
OR SO2[tiab] OR "sulfur dioxide"[tiab] OR CO[tiab] OR "carbon monoxide"[tiab]
OR "industrial emissions"[tiab] OR "traffic-related air pollution"[tiab]
OR "environmental exposure*"[tiab] OR "environmental factor*"[tiab]
OR "environmental determinant*"[tiab] OR "environmental condition*"[tiab]
OR "urban heat island"[tiab] OR "built environment"[tiab]
OR ventilation[tiab] OR overcrowding[tiab]
)
```

Environmental<br><br>exposures/factors	"Mycobacterium tuberculosis"[Mesh] OR "Tuberculosis"[Mesh] OR "Tuberculosis, Pulmonary"[Mesh]<br>OR "Mycobacterium tuberculosis"[tiab] OR tuberculosis[tiab] OR TB[tiab]<br>OR "pulmonary tuberculosis"[tiab] OR "extrapulmonary tuberculosis"[tiab]<br>OR "disseminated tuberculosis"[tiab]

```
OR "drug-resistant tuberculosis"[tiab] OR "drug
resistant TB"[tiab]
OR "multidrug-resistant tuberculosis"[tiab] OR "MDR
TB"[tiab] OR "MDR-TB"[tiab]
OR "extensively drug-resistant tuberculosis"[tiab] OR
"XDR TB"[tiab] OR "XDR-TB"[tiab]
OR "rifampicin-resistant TB"[tiab] OR "RR-TB"[tiab]
OR "tuberculous meningitis"[tiab] OR "miliary
tuberculosis"[tiab] OR "TB meningitis"[tiab]
OR MTB[tiab] OR "Mycobacterium tuberculosis
complex"[tiab]
)
AND
(
"Climate Change"[Mesh] OR "Environmental
Exposure"[Mesh]
OR "climate change"[tiab] OR "climate
variability"[tiab]
```

AND ‘commuting’, ‘public transport’, ‘transit crowding’, ‘transit hub’,

‘school attendance’, ‘workplace attendance’, ‘migration’, ‘displacement’

SCOPUS (Elsevier)

Uses **TITLE-ABS-KEY**

```
(
TITLE-ABS-KEY("Mycobacterium tuberculosis" OR tuberculosis OR TB
OR "pulmonary tuberculosis" OR "extrapulmonary tuberculosis"
OR "disseminated tuberculosis"
OR "drug-resistant tuberculosis" OR "drug resistant TB"
OR "multidrug-resistant tuberculosis" OR "MDR TB" OR "MDR-TB"
OR "extensively drug-resistant tuberculosis" OR "XDR TB" OR "XDR-TB"
OR "rifampicin-resistant TB" OR "RR-TB"
OR "tuberculous meningitis" OR "miliary tuberculosis" OR "TB meningitis"
OR MTB OR "Mycobacterium tuberculosis complex")
)
AND
(
TITLE-ABS-KEY("climate change" OR "climate variability"
OR temperature OR "temperature variation*"
OR heat OR cold
OR heatwave* OR "heat wave*" OR "extreme temperature*"
OR "extreme weather*" OR "weather variability"
OR season*
OR "relative humidity" OR "absolute humidity" OR "dew point"
OR rainfall OR precipitation
OR "air pollution" OR "air quality"
OR "particulate matter" OR PM2.5 OR PM10
OR ozone OR O3 OR NO2 OR "nitrogen dioxide"
OR SO2 OR "sulfur dioxide" OR CO OR "carbon monoxide"
OR "industrial emissions" OR "traffic-related air pollution"
OR "environmental exposure*" OR "environmental factor*"
```

```
OR "environmental determinant*" OR "environmental condition*"
OR "urban heat island" OR "built environment"
OR ventilation OR overcrowding
OR drought OR "wildfire smoke" OR "dust storm*")
)
```

Web of Science Core Collection (Clarivate Analytics)

Uses **TS=Topic (Title + Abstract + Author Keywords)**

```
TS=(
"Mycobacterium tuberculosis" OR tuberculosis OR TB
OR "pulmonary tuberculosis" OR "extrapulmonary tuberculosis"
OR "disseminated tuberculosis"
OR "drug-resistant tuberculosis" OR "drug resistant TB"
OR "multidrug-resistant tuberculosis" OR "MDR TB" OR "MDR-TB"
OR "extensively drug-resistant tuberculosis" OR "XDR TB" OR "XDR-TB"
OR "rifampicin-resistant TB" OR "RR-TB"
OR "tuberculous meningitis" OR "miliary tuberculosis" OR "TB meningitis"
OR MTB OR "Mycobacterium tuberculosis complex"
)
AND
TS=(
"climate change" OR "climate variability"
OR temperature OR "temperature variation*"
OR heat OR cold
OR heatwave* OR "heat wave*" OR "extreme temperature*"
OR "extreme weather*" OR "weather variability"
OR season*
OR "relative humidity" OR "absolute humidity" OR "dew point"
OR rainfall OR precipitation
OR "air pollution" OR "air quality"
OR "particulate matter" OR PM2.5 OR PM10
OR ozone OR O3 OR NO2 OR "nitrogen dioxide"
OR SO2 OR "sulfur dioxide" OR CO OR "carbon monoxide"
OR "industrial emissions" OR "traffic-related air pollution"
OR "environmental exposure*" OR "environmental factor*"
OR "environmental determinant*" OR "environmental condition*"
OR "urban heat island" OR "built environment"
OR ventilation OR overcrowding
OR drought OR "wildfire smoke" OR "dust storm*"
)
```

Ovid MEDLINE (EBSCO / Ovid)

Uses **MeSH + .ti,ab.**

```
(
Mycobacterium tuberculosis/ OR Tuberculosis/ OR Tuberculosis, Pulmonary/
OR Mycobacterium tuberculosis.ti,ab. OR tuberculosis.ti,ab. OR TB.ti,ab.
OR pulmonary tuberculosis.ti,ab. OR extrapulmonary tuberculosis.ti,ab.
OR disseminated tuberculosis.ti,ab.
```

```
OR drug-resistant tuberculosis.ti,ab. OR drug resistant TB.ti,ab.
OR multidrug-resistant tuberculosis.ti,ab. OR MDR TB.ti,ab. OR MDR-TB.ti,ab.
OR extensively drug-resistant tuberculosis.ti,ab. OR XDR TB.ti,ab. OR XDR-
TB.ti,ab.
OR rifampicin-resistant TB.ti,ab. OR RR-TB.ti,ab.
OR tuberculous meningitis.ti,ab. OR miliary tuberculosis.ti,ab. OR TB
meningitis.ti,ab.
OR MTB.ti,ab. OR Mycobacterium tuberculosis complex.ti,ab.
)
AND
(
Climate Change/ OR Temperature/ OR Humidity/ OR Air Pollution/ OR Air
Quality/ OR Environmental Exposure/
OR "climate change".ti,ab. OR "climate variability".ti,ab.
OR temperature.ti,ab. OR "temperature variation*".ti,ab.
OR heat.ti,ab. OR cold.ti,ab.
OR heatwave*.ti,ab. OR "heat wave*".ti,ab. OR "extreme temperature*".ti,ab.
OR "extreme weather*".ti,ab. OR "weather variability".ti,ab.
OR season*.ti,ab.
OR "relative humidity".ti,ab. OR "absolute humidity".ti,ab. OR "dew
point".ti,ab.
OR rainfall.ti,ab. OR precipitation.ti,ab.
OR "air pollution".ti,ab. OR "air quality".ti,ab.
OR "particulate matter".ti,ab. OR PM2.5.ti,ab. OR PM10.ti,ab.
OR ozone.ti,ab. OR O3.ti,ab. OR NO2.ti,ab. OR "nitrogen dioxide".ti,ab.
OR SO2.ti,ab. OR "sulfur dioxide".ti,ab. OR CO.ti,ab. OR "carbon
monoxide".ti,ab.
OR "industrial emissions".ti,ab. OR "traffic-related air pollution".ti,ab.
OR "environmental exposure*".ti,ab. OR "environmental factor*".ti,ab.
OR "environmental determinant*".ti,ab. OR "environmental condition*".ti,ab.
OR "urban heat island".ti,ab. OR "built environment".ti,ab.
OR ventilation.ti,ab. OR overcrowding.ti,ab.
OR drought.ti,ab. OR "wildfire smoke".ti,ab. OR "dust storm*".ti,ab.
)
```

Supplementary tables

Supplementary table 1. Development of the search strategy

Search term category	Search terms
General TB terms	‘tuberculosis’, ‘TB’, ‘MTB’, ‘*Mycobacterium tuberculosis*’, ‘*Mycobacterium tuberculosis* complex’
Forms of TB	‘pulmonary tuberculosis’, ‘extrapulmonary tuberculosis’, ‘disseminated tuberculosis’, ‘tuberculous meningitis’, ‘miliary tuberculosis’, ‘TB meningitis’
Drug resistance	‘drug-resistant tuberculosis’, ‘drug resistant TB’, ‘MDR TB’, ‘MDR-TB’, ‘multidrug-resistant tuberculosis’, ‘XDR TB’, ‘XDR-TB’, ‘extensively drug-resistant tuberculosis’, ‘RR-TB’, ‘rifampicin-resistant TB’
Climate and weather	‘climate change’, ‘climate variability’, ‘temperature’, ‘cold’, ‘heat’, ‘heatwaves’, ‘heat wave’, ‘*cold temperature*’, ‘temperature variation’, ‘*weather variability*’,

	'extreme weather', 'extreme temperature', *'season*', 'dew point', 'absolute humidity', 'rainfall', 'precipitation'
Air quality/pollution	'air pollution', 'air quality', '$PM_{2.5}$', 'PM_{10}', 'ozone', 'NO_2', 'SO_2', 'CO', 'particulate matter', 'industrial emissions', 'traffic-related air pollution', 'wildfire smoke', 'dust storms'
Environmental exposures/factors	'environmental exposure', 'environmental factor', 'environmental determinant', *'environmental condition*', 'built environment', 'urban heat island', 'ventilation', 'overcrowding', 'crowding', 'commuting', 'public transport', 'transit crowding', 'transit hub', 'school attendance', 'workplace attendance', 'migration', 'displacement', 'drought'

Supplementary table 2. Inclusion and exclusion criteria used for screening

Category	Include	Exclude
Population	Human TB (pulmonary, extrapulmonary, disseminated, TB meningitis, miliary TB); drug-resistant TB (MDR-TB, XDR-TB, RR-TB)	Non-human TB; TB mentioned incidentally/not primary focus; non-tuberculous mycobacteria
Exposure	Environmental/climatic factors: climate/weather (temperature, heat, cold, rainfall, humidity, extreme events, seasonality), air quality/pollution ($PM_{2.5}$, PM_{10}, NO_2, SO_2, CO, ozone, traffic/industrial emissions, wildfire smoke, dust), other factors (ventilation, overcrowding, urban heat islands, migration, school/work attendance, commuting, drought)	Minimal/no environmental/climatic assessment; only social/economic determinants
Study Type	Primary research (observational/experimental); meta-analyses	Conference abstracts, reviews, editorials, letters, opinion pieces, non-peer-reviewed publications
Publication	Peer-reviewed journals; English language only	Non-peer-reviewed; non-English publications
Data Availability	Full text available	Full text unavailable

Supplementary table 3. The country-specific author prevalence within the profile of publications on environmental factors linked to TB

Country	Frequency
China	427
United States of America	77
United Kingdom	27
Japan	24
South Africa	21
India	18
Iran	17
Brazil	11
Malaysia	10
Romania	9
Australia	9
Peru	8
Spain	8
South Korea	6
Vietnam	6
Colombia	5
Canada	4
Kenya	4
Mexico	4
Morocco	4
Switzerland	4
Uganda	4
Brunei	3
France	3
Indonesia	3
Malawi	3
Mongolia	3
Afghanistan	2
Ethiopia	2
Italy	2
Pakistan	2
Tanzania	2
Bolivia	1
Denmark	1

Georgia	1
Ghana	1
Hungary	1
Ireland	1
Nigeria	1
Russia	1
Syria	1
Zambia	1
Zimbabwe	1

Supplementary table 4. The top 20 most cited articles from the 124 screened articles taken from Bibliometrix

**Author, year**	Journal	Title	Study site	Total Citations	Main findings
Martineau et al., 2011	PNAS	Reciprocal seasonal variation in vitamin D status and tuberculosis notifications in Cape Town, South Africa	Cape Town South Africa	165	• TB notifications lowest in Apr–Jun and highest in Oct–Dec.<br>• Vitamin D deficiency associated with increased TB risk – HIV + and HIV -<br>• Seasonal patterns in vitamin D and TB incidence - causal relationship.
Sita-Lumsden et al., 2007	Thorax	Reactivation of tuberculosis and vitamin D deficiency: the contribution of diet and exposure to sunlight	London, United Kingdom	109	• TB patients - significantly lower Vitamin D levels than matched contacts.<br>• TB patients did not show normal seasonal variation in Vitamin D levels.<br>• Abnormal Vitamin D metabolism contributes to TB susceptibility.
Smith et al., 2016	Environmental Health Perspectives	Air Pollution and Pulmonary Tuberculosis: A Nested Case–Control Study among Members of a Northern California Health Plan	Northern California, USA	97	• Positive associations between CO and NO_2 exposure and pulmonary TB.<br>• Residents in northern California exposed to higher air pollution had increased TB risk.<br>• Results support air pollution as a TB risk factor.
Andrews et al., 2013	American Journal of Epidemiology	Modeling the Role of Public Transportation in Sustaining Tuberculosis Transmission in South Africa	Cape Town, South Africa	90	• TB infection risk highest among minibus taxi commuters due to poor ventilation and crowding.<br>• Annual risk of TB infection was projected to be 3.5%-5.0% and was highest among minibus taxi commuters.<br>• Public transportation - key role in urban TB spread.
Nakaji et al., 2004	European Journal of Epidemiology	Seasonal changes in mortality rates from main causes of death in Japan (1970–1999)	Japan	89	• TB and respiratory diseases peaked in winter.<br>• Seasonal influence on TB and respiratory disease declined - 1970 to 1999.<br>• Reducing seasonally driven diseases could improve overall life expectancy.
Kodgule & Salvi, 2012	Current Opinion in	Exposure to biomass smoke as a cause for	global	79	• Biomass smoke exposure increases respiratory infections, including TB.

	Allergy and Clinical Immunology	airway disease in women and children			• Women and children are the most affected populations.<br>• Biomass smoke may pose a greater COPD risk than tobacco smoking.
Huang et al., 2020	Environmental Research	Association between short-term exposure to ambient air pollutants and the risk of tuberculosis outpatient visits: A time-series study in Hefei, China	Hefei, China	73	• Increases in $PM_{2.5}$, PM_{10}, and O_3 were associated with higher TB risk.<br>• $PM_{2.5}$ remained significant compared to other pollutants.<br>• Air pollution impacts on TB were similar in warm and cold seasons.
Liu et al., 2015	AIDS	Tuberculosis incidence rate and risk factors among HIV-infected adults with access to antiretroviral therapy in Tanzania	Tanzania	70	• TB risk was highest in the months following ART initiation.<br>• Male sex and poor nutritional status increased TB risk.<br>• Nutritional support and monitoring are critical alongside ART.
Sarkar et al., 2012	Journal of Immunology	Suppression of the NF-κB Pathway by Diesel Exhaust Particles Impairs Human Antimycobacterial Immunity	New Jersey, USA	69	• DEP suppressed key pro-inflammatory immune responses to *M. tuberculosis*.<br>• DEP altered Toll-like receptor and immune gene expression pathways.<br>• Air pollution weakens host defences against TB infection.
Rao et al., 2016	Infectious Diseases of Poverty	Spatial transmission and meteorological determinants of tuberculosis incidence in Qinghai Province, China: a spatial clustering panel analysis	China	69	• TB incidence showed strong spatial clustering across counties.<br>• TB incidence associated with temperature, precipitation, and wind speed.<br>• Cold, dry, and windy regions had higher TB incidence.
Naranbat et al., 2009	European Respiratory Journal	Seasonality of tuberculosis in an Eastern–Asian Country with an Extreme Continental Climate	Mongolia	68	• TB notifications peaked in April and were lowest in Oct – Dec.<br>• Seasonal patterns were consistent across age, sex, and location.<br>• Temperature likely influences TB transmission and delayed diagnosis rather than healthcare access.
Sun et al., 2015	International Journal of Environmental Research and Public Health	A Spatial, Social and Environmental Study of Tuberculosis in China Using Statistical and GIS Technology	China	67	• TB prevalence significantly associated with multiple latent factors - altitude, longitude, climate, and education burden.<br>• Some factors (employment, population density, air quality) showed effects on TB prevalence, while health services and unemployment had limited impact.<br>• The influence of each factor varied geographically, highlighting the need for region-specific TB control strategies.
Guo et al., 2017	Epidemiology and Infection	Spatiotemporal analysis of tuberculosis incidence and its associated factors in mainland China	China	61	• TB incidence showed strong spatial clustering with two main hot spots and a consistent late-winter seasonal peak.<br>• Higher TB incidence was associated with a larger elderly population, while higher

					GDP per capita and higher male:female ratio linked to lower incidence.<br>• Meteorological, demographic, and socio-economic factors all influenced TB, supporting targeted regional control strategies.
Friis et al., 2008	Journal of Nutrition	Hypovitaminosis D is common among pulmonary tuberculosis patients in Tanzania but is not explained by the acute phase response	Tanzania	57	• Vitamin D<br>○ highest during the harvest season (May–July)<br>○ lower in TB positive patients compared TB negative patients<br>• Consumption of large freshwater fish, BMI, and serum transferrin receptor positively predicted Vitamin D levels; HIV status did not.
Yang et al., 2020	International Journal of Infectious Diseases	A study on the relationship between air pollution and pulmonary tuberculosis based on the general additive model in Wulumuqi, China	China	49	• Exposure to $PM_{2.5}$, SO_2, NO_2, and CO was associated with increased TB incidence after 3 months; PM10 exposure showed effects after 9 months.<br>• Single-pollutant models indicated that increases in $PM_{2.5}$, PM_{10}, SO_2, NO_2, CO, and O_3 all raised TB case numbers, with CO having the largest effect.<br>• The optimal multi-pollutant model included PM_{10} and NO_2, highlighting air pollution as a key risk factor for TB and a need for further mechanistic studies.
Yao et al., 2019	Environment International	Ambient air pollution exposures and risk of drug-resistant tuberculosis	China	48	• Exposure to $PM_{2.5}$, PM_{10}, O_3, and CO was associated with higher risks of mono-drug, poly-drug, and multi-drug resistant TB.<br>• $PM_{2.5}$ showed the strongest effect after ~540 days, O_3 after ~180 days, and PM_{10}/CO between 90–540 days of exposure.<br>• Both single- and multi-pollutant models confirmed that ambient air pollution contributes to the development of DR-TB.
Ibironke et al., 2019	International Journal of Environmental Research and Public Health	Urban Air Pollution Particulates Suppress Human T-Cell Responses to *Mycobacterium tuberculosis*	New Jersey, USA	45	• $PM_{2.5}$ exposure<br>○ reduced PBMC capacity to control Mtb growth<br>○ suppressed early T cell activation (CD69)<br>○ decreased pro-inflammatory cytokines (IFN-γ in $CD3^+$ cells, TNF-α in $CD3^+$ and $CD14^+$ cells)<br>○ Increased anti-inflammatory IL-10<br>○ Impairs antimycobacterial T cell responses, weakening host defence against TB.
Azeez et al., 2016	International Journal of Environmental	Seasonality and Trend Forecasting of Tuberculosis Prevalence Data in Eastern Cape, South	Eastern Cape, South Africa	45	• Both models could predict TB incidence, but the combined SARIMA-NNAR model performed better, with lower AIC and BIC values.

	Research and Public	Africa, Using a Hybrid Model	• The SARIMA-NNAR model forecasted a slight seasonal increase in TB incidence, highlighting seasonal patterns.<br>• Results emphasize the need for targeted interventions during periods of high transmission, including festival peaks and co-infection with HIV.

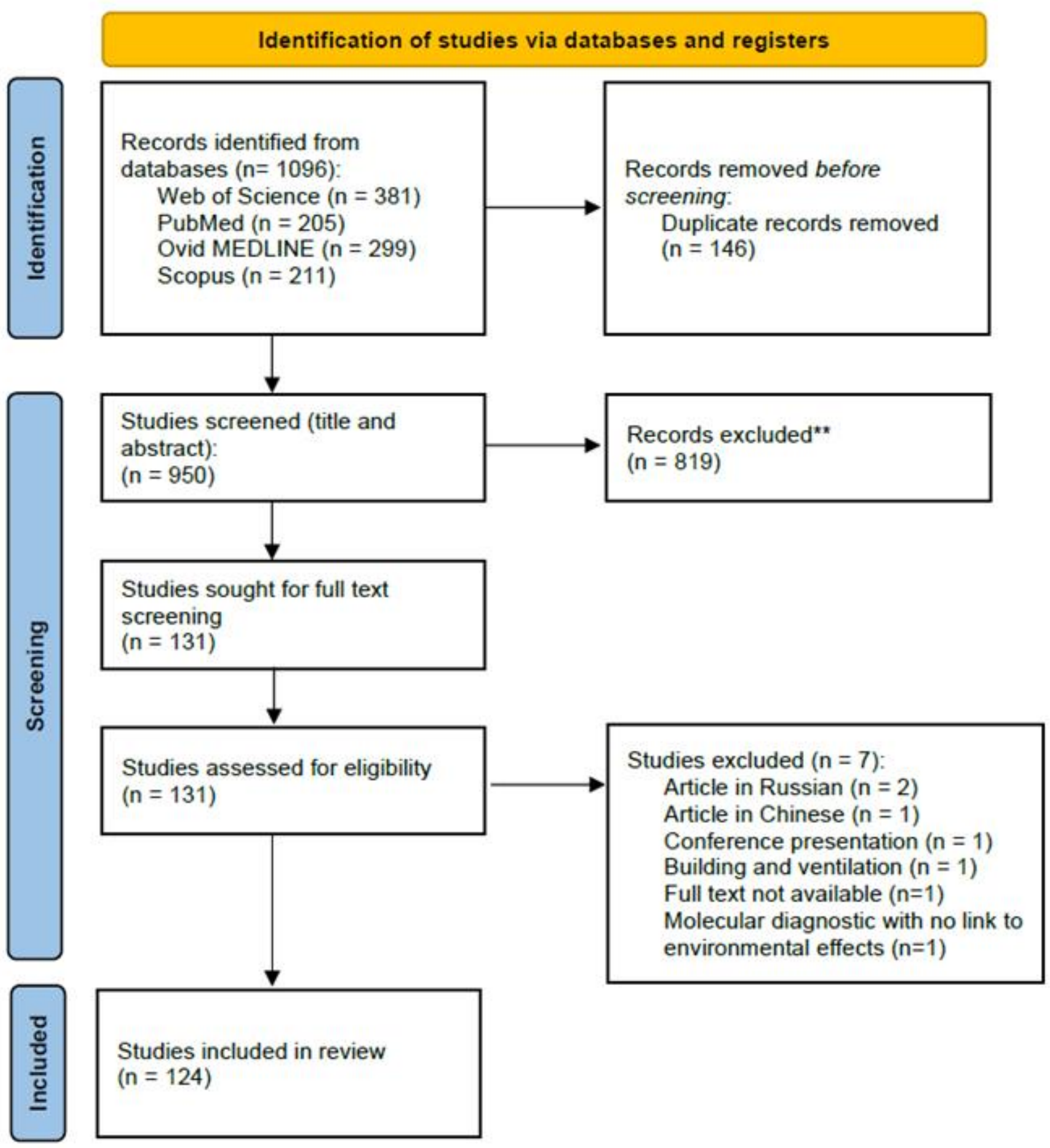

Supplementary figure 1. PRISMA diagram.

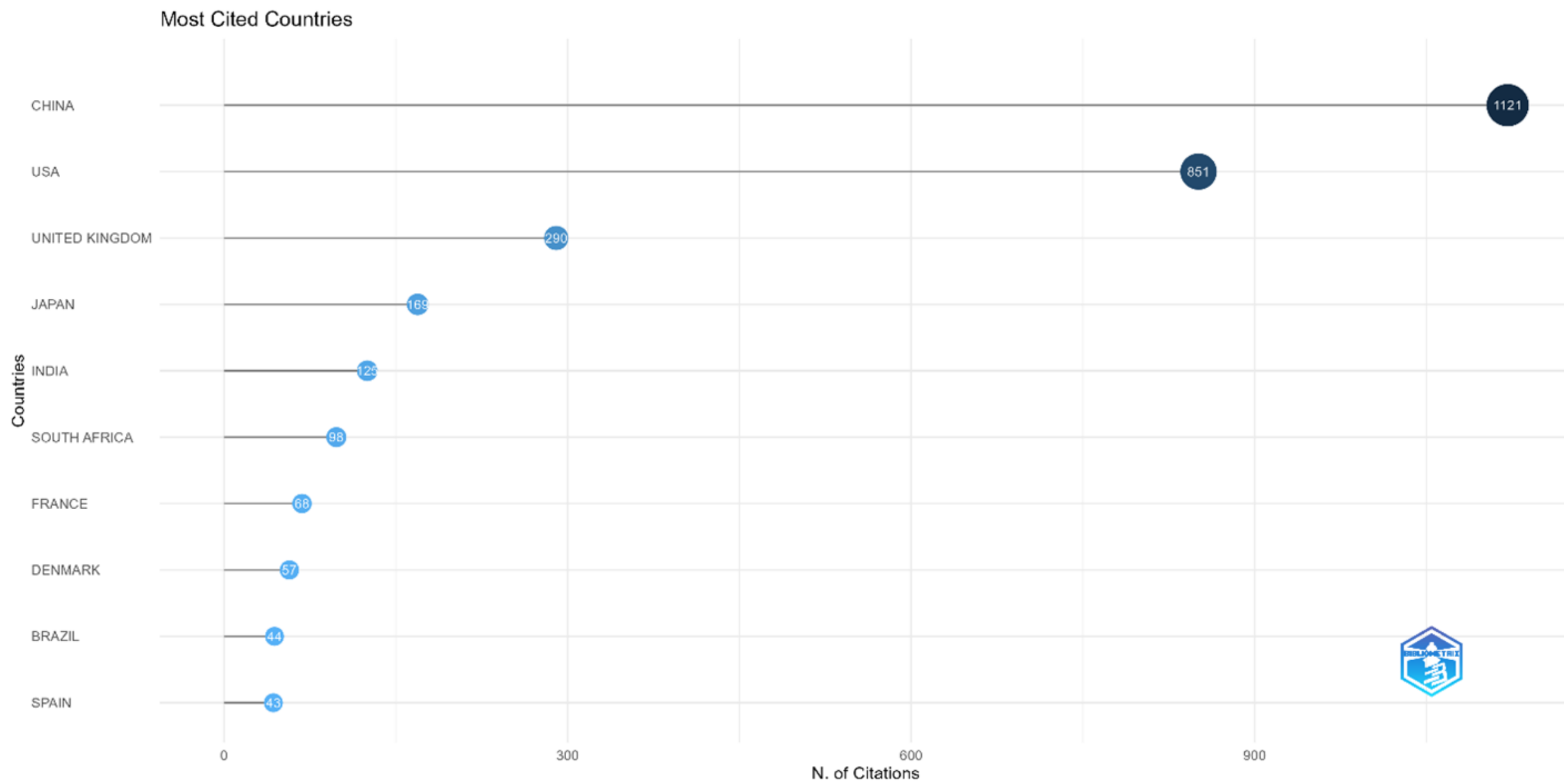

Supplementary figure 2. Most cited countries. The chart illustrates the most cited countries in research, with China leading with over 1121 citations, followed by the USA and the United Kingdom (UK). Other notable countries include Japan, India, and South Africa, each with significant contributions.

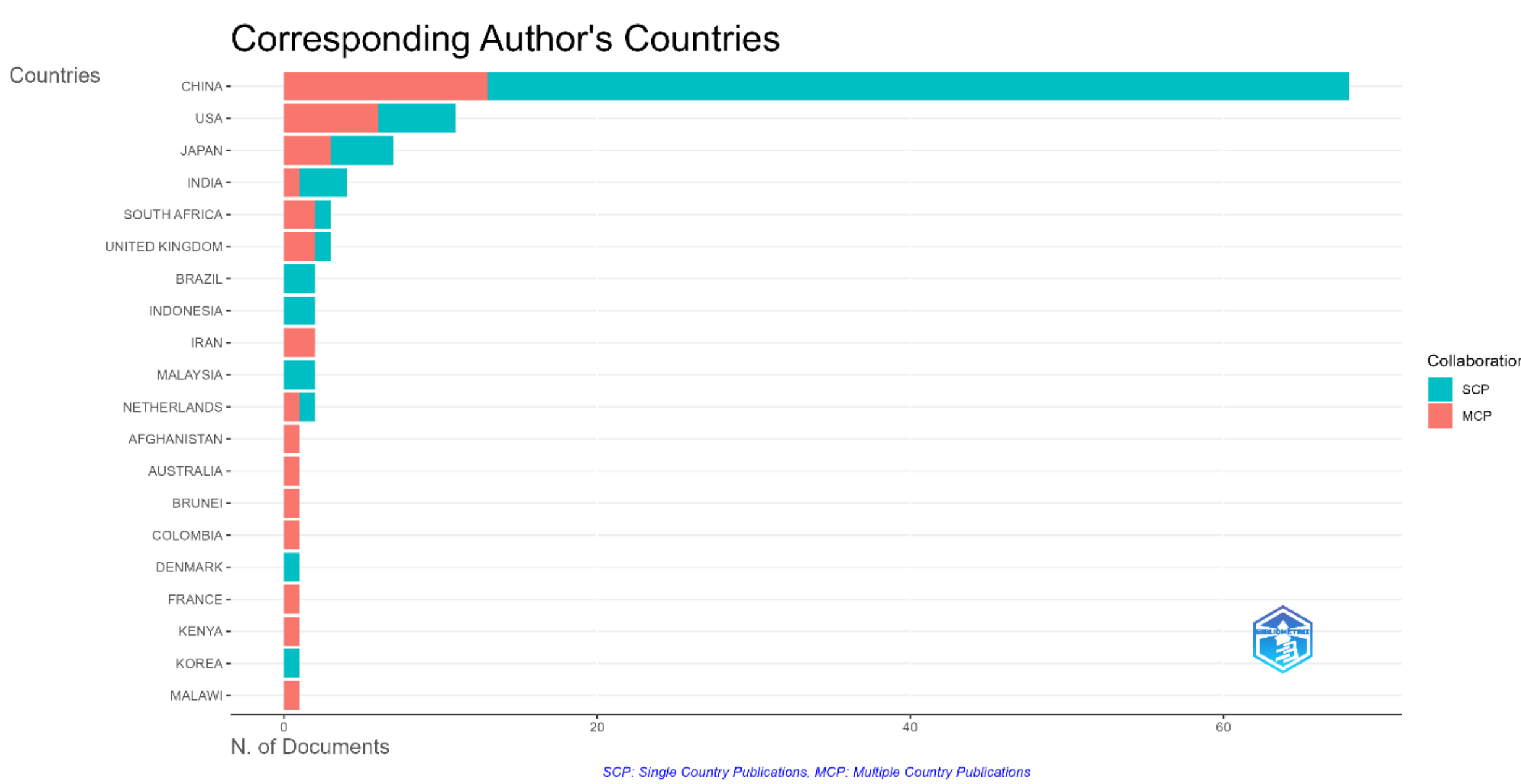

Supplementary figure 3. The corresponding author country affiliations and their contributions to local and global research in the studies screened. The image shows single country publications (SCP) in red and multi-country publications (MCP) in blue

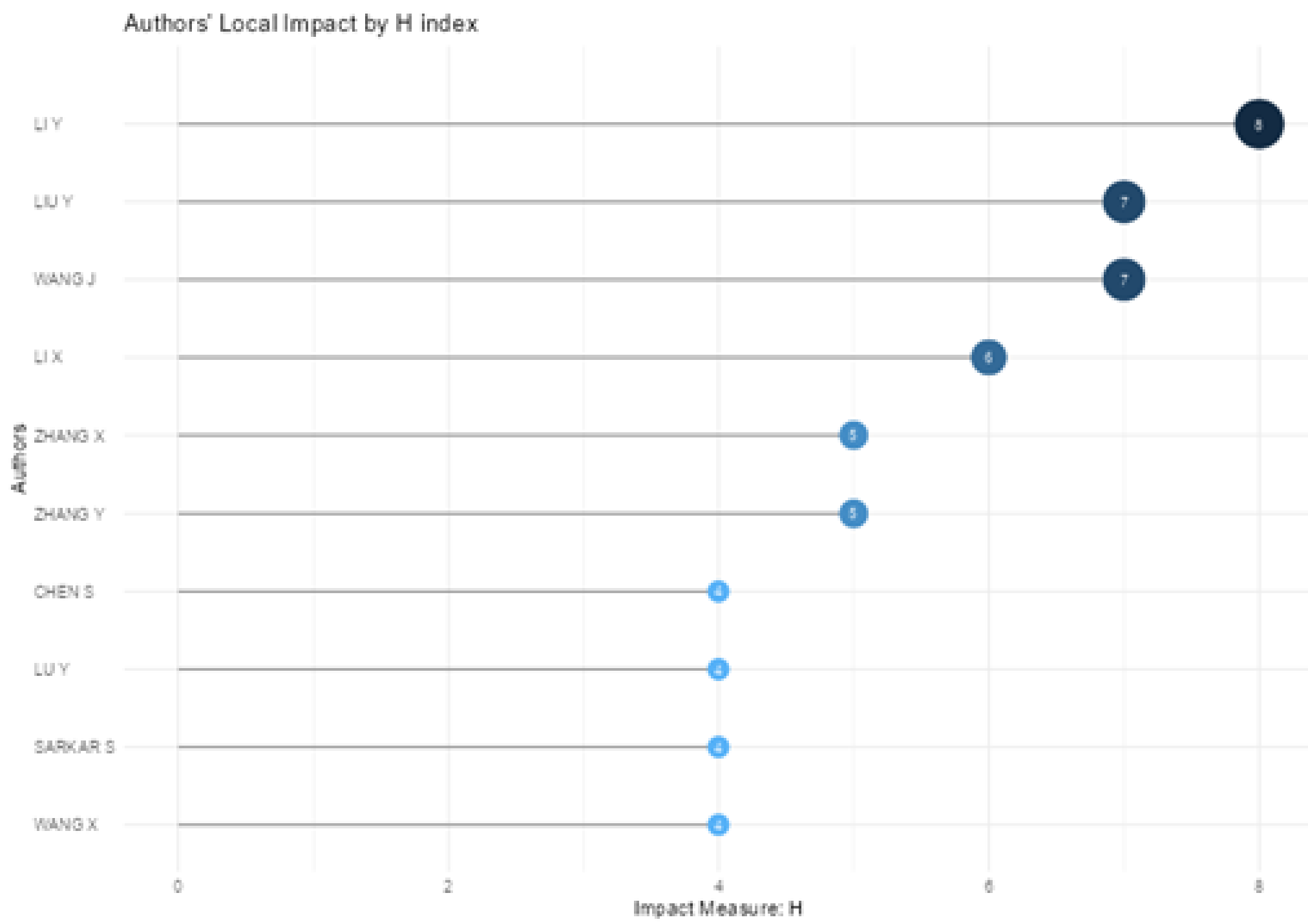

Supplementary figure 4. Authors local contributions by H-index

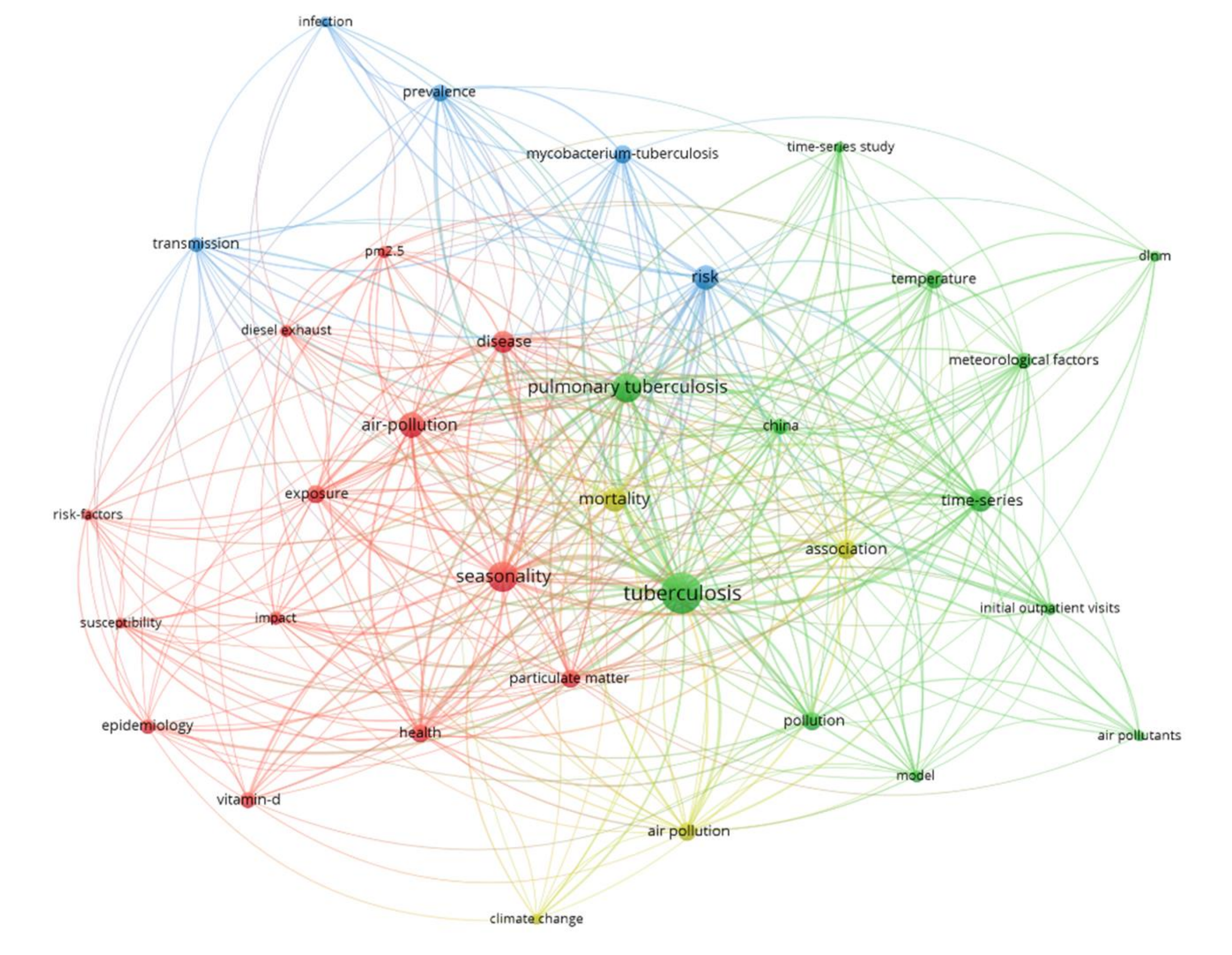

Supplementary figure 5. Keyword map generated from Vosviewer strong clusters and highlighting a critical shift toward TB exposure science. The green and red clusters, which link $PM_{2.5}$ and meteorological modelling to infection risk, suggest that the research community no longer views TB in isolation from environmental exposure.

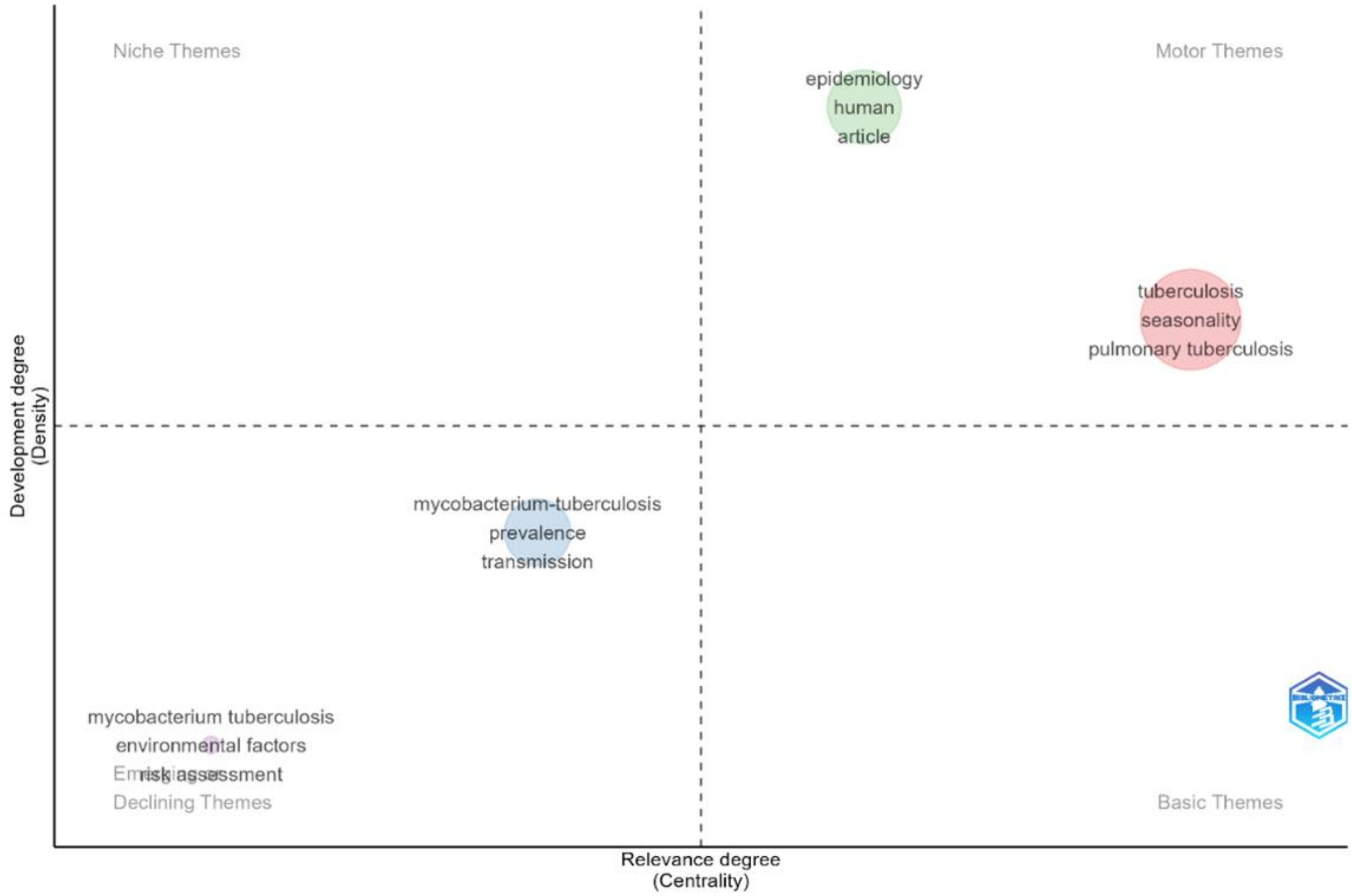

Supplementary figure 6. Keyword analysis generated by Bibliometrix software. The four quadrants represent the degree of development versus the relevance of themes to the overall topic. The bottom left corner is emerging and declining themes as these themes are not well established and include risk assessments of TB in relation to the environment. The top right-hand corner has the most developed themes of TB in relation to seasonality human epidemiology.
